# Correlation Between Distance From Lesions to Pleura and Complications in CT‐Guided Lung Biopsy: A Cross‐Sectional Study in Vietnam

**DOI:** 10.1111/1759-7714.70286

**Published:** 2026-04-27

**Authors:** Thanh Dinh Le, Thuan Nguyen Huynh, Thanh Chi Nguyen, Hau Thi Tran, Nguyen Vo Cong Do, Khang Quang Le, Son Thai Tran, Thanh Toan Vo

**Affiliations:** ^1^ Thong Nhat Hospital Ho Chi Minh City Vietnam; ^2^ University of Medicine and Pharmacy Ho Chi Minh City Vietnam; ^3^ Buon Ma Thuot Medical University Hospital Buon Ma Thuot Vietnam

**Keywords:** biopsy cores, complications, CT‐guided, lung biopsy, traversed lung parenchymal distance

## Abstract

**Introduction:**

CT‐guided transthoracic needle biopsy (CT‐TTNB) is an essential diagnostic procedure but is associated with complications such as pneumothorax and pulmonary hemorrhage. The distance from the lesion to the pleura (traversed parenchymal distance) is widely recognized as a major risk factor. This study aims to evaluate the correlation between the traversed lung parenchymal distance and post‐CT‐TTNB complications, and to identify independent predictors for these complications.

**Methods:**

A retrospective cross‐sectional study was conducted on 226 patients who underwent CT‐TTNB. Data on demographics, lesion characteristics, and procedure‐related factors (traversed parenchymal distance, number of biopsy samples) were collected. Multivariable logistic regression analysis was used to identify independent predictors for pneumothorax and pulmonary hemorrhage.

**Results:**

The overall complication rate was 47.35%, with pneumothorax at 29.2% and pulmonary hemorrhage at 32.74%. Multivariable analysis showed that statistically significant independent predictors for pneumothorax were male gender (adjusted odds ratio [aOR] = 3.78; 95% confidence interval [CI]: 1.81–7.89; *p* < 0.001) and the number of biopsy samples (aOR = 0.65; 95% CI: 0.45–0.94; *p* = 0.022). The traversed lung parenchymal distance was not a statistically significant independent predictor for either pneumothorax (*p* = 0.251) or pulmonary hemorrhage.

**Conclusions:**

In this study population, male gender and the number of biopsy samples were significantly associated with the risk of pneumothorax. The traversed lung parenchymal distance did not emerge as an independent predictor in the multivariable model. These findings challenge the traditional view and emphasize the need for a multifactorial risk assessment model rather than relying on a single parameter.

## Graphical abstract

In this study, multivariable analysis revealed that male gender and biopsy sample quantity independently predict pneumothorax following CT‐guided lung biopsy, whereas traversed parenchymal distance does not. These findings challenge traditional views, emphasizing the necessity of multifactorial risk assessment over relying on a single parameter.

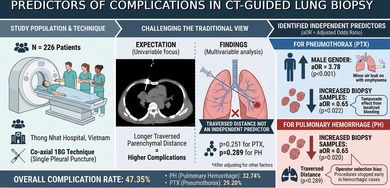

## 1 Introduction

CT‐guided transthoracic needle biopsy (CT‐TTNB) has become a minimally invasive method in modern clinical practice. This technique plays a crucial role in diagnosing the histopathological nature of lung lesions, especially peripheral pulmonary nodules or masses suspected of malignancy. With its ability to precisely locate the needle path, CT‐TTNB offers high diagnostic efficacy, with sensitivity and specificity in identifying malignancy often exceeding 90%—thereby providing a basis for timely and appropriate treatment planning for patients [1].

Despite its high efficacy, lung biopsy remains an invasive procedure with potential risks of complications. Among these, pneumothorax (PTX) and pulmonary hemorrhage (PH) are the two most common complications reported in the medical literature [1, 2]. International systematic reviews and meta‐analyses have reported a very wide range of complication rates, reflecting differences in patient populations, procedural techniques, and operator experience among centers. Specifically, the rate of PTX ranges from 8% to 45%, while the rate of PH can range from 3% to 55% [1]. Rarer but more serious complications include air embolism, severe hemoptysis, and tumor cell seeding along the biopsy needle track [2].

To optimize patient safety, identifying risk factors associated with complications is crucial. The world literature has identified a range of risk factors, including patient‐related factors (older age, history of chronic obstructive pulmonary disease—COPD), lesion‐related factors (small size, location in the lower lobe), and technical factors (number of pleural passes, needle angle relative to the pleura) [3]. The distance from the lesion to the pleura, or the length of the needle path through the lung parenchyma, is one of the most important and consistent predictors for both PTX and PH [2, 3]. The theoretical basis for this association is that a longer needle path through the lung parenchyma increases the probability of damaging alveolar structures, small bronchioles, and blood vessels, leading to air leakage into the pleural space or bleeding into the alveoli.

However, previous research has primarily relied on univariate analyses to assess the role of traversed parenchymal distance, which may lead to an overestimation of its role by not adjusting for potential confounding factors. The independent role of this distance when considered simultaneously with other risk factors in a multivariable model has not been fully clarified. Furthermore, data on these risk factors, especially studies with multivariable analysis, are very limited in the Vietnamese patient population.

Therefore, this study was conducted with two main objectives: (1) to evaluate the correlation between the traversed lung parenchymal distance and the risk of complications after CT‐TTNB in a Vietnamese patient population; and (2) to identify independent predictors for these complications through multivariable analysis.

## 2 Method

### 2.1 Study Design and Population

We conducted a cross‐sectional study on patients who underwent CT‐guided transthoracic lung biopsy at Thong Nhat Hospital. The analysis included a total of 226 consecutive patients from May 2023 to May 2025. This study complied with the ethical standards outlined by the Declaration of Helsinki. Ethical approval from the Thong Nhat Hospital was obtained (Approval No. 738/BVTN 07/05/2025).

### 2.2 Inclusion Criteria

All patients with focal lung lesions requiring histopathological diagnosis who were indicated for CT‐guided lung biopsy.

### 2.3 Exclusion Criteria

Patients with severe uncorrectable coagulopathy, acute respiratory failure, or incomplete electronic medical records lacking imaging data required to measure the traversed parenchymal distance.

### 2.4 Data Collection

Data were retrospectively collected from electronic medical records and the Picture Archiving and Communication System (PACS). To minimize technical variability, all procedures were performed using a standardized 18‐G semiautomatic biopsy needle by a dedicated team of interventional radiologists with more than 5 years of experience. All CT‐guided biopsies were performed using a co‐axial technique to minimize pleural trauma, involving a single pleural puncture with a guiding needle through which multiple tissue samples were obtained. The collected information included demographic characteristics (age, gender); lesion characteristics, specifically distance from the lesion to the pleura (traversed lung parenchymal distance), which was measured on pre‐procedural CT images as the actual length of the needle track from the pleural entry point to the lesion boundary along the planned biopsy path; procedure‐related factors, including the number of biopsy samples obtained; and postprocedural complications. The occurrence of complications, including PTX, PH, and hemoptysis was recorded based on immediate post‐biopsy CT images and clinical follow‐up within 24 h.

### 2.5 Statistical Analysis

Data were analyzed using statistical software SPSS (Chicago, IL, USA). Continuous variables were described as mean ± standard deviation (SD) for normally distributed data and as median with interquartile range (IQR) for non‐normally distributed data, while categorical variables were expressed as frequencies and percentages. The chi‐squared test was used to compare complication rates among different traversed parenchymal distance groups, while Fisher's exact test was applied when the expected cell count was less than 5. Univariate and multivariable logistic regression analyses were performed to identify independent predictors for PTX and PH. Variables were selected for multivariable logistic regression based on clinical relevance or a *p*‐value < 0.20 in univariable analysis to avoid premature exclusion of potential confounders, such as lesion‐to‐pleura distance. This approach ensures that potential confounders, such as the traversed lung parenchymal distance, are not prematurely excluded from the model despite not reaching statistical significance in simple correlation tests. Odds ratios (OR) and adjusted odds ratios (aOR) with their 95% confidence intervals (CI) were calculated. Statistical significance was set at *p* < 0.05.

Regarding sample size, all eligible consecutive patients during the study period were included without a formal a priori power calculation. To address missing data, cases with incomplete imaging records that prevented accurate measurement of the traversed parenchymal distance were excluded from the final analysis. Inter‐rater reliability for distance measurements was not formally assessed; however, measurement bias was minimized by utilizing a standardized measurement protocol performed by a dedicated team of experienced interventional radiologists.

## 3 Results

### 3.1 General Characteristics

The study included 226 patients, of whom the majority were male with 149 cases (65.93%). The average age of the patients was 66.92 ± 11.23 years. Subgroup analysis revealed that the mean age for male patients was 66.89 ± 10.63 years, while for female patients, it was 66.97 ± 12.39 years, with no statistically significant difference between the two groups (*p* > 0.05). All procedures were performed using a co‐axial biopsy technique, which involved a single pleural puncture followed by the collection of multiple tissue samples. The average number of biopsy samples taken per procedure was 4.45 ± 0.99.

### 3.2 Complication Rates and Lesion Characteristics

**FIGURE 1:**
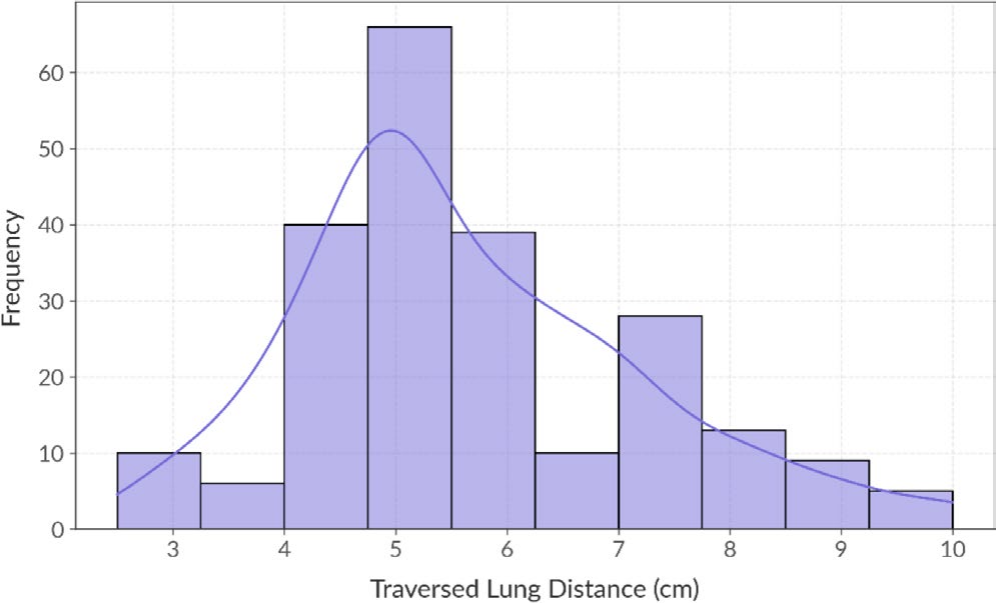
Distribution of traversed lung distance.

Regarding postprocedural outcomes, the overall complication rate observed in this study was 47.35%. As detailed in Table 1, PH was the most prevalent complication, affecting 74 patients (32.74%), followed by PTX in 66 cases (29.2%). Other complications, including hemoptysis and minor adverse events, occurred less frequently at rates of 1.33% and 0.88%, respectively. In terms of lesion accessibility, the distribution of the traversed lung parenchymal distance is illustrated in Figure 1. The study population had a mean traversed distance of 5.66 ± 1.55 cm. Notably, the majority of the patients (55.31%) exhibited a distance ranging from 4 to 6 cm.

**TABLE 1:** Postprocedural complication rates.

Complication	Complication rates, *N* (%)
Any complication	107 (47.35)
Pulmonary hemorrhage	74 (32.74)
Pneumothorax	66 (29.20)
Hemoptysis	3 (1.33)
Other complications	2 (0.88)

### 3.3 Correlation and Predictors of Complications

The analysis of the correlation between the traversed parenchymal distance and complication types, summarized in Table 2, revealed a statistically significant association with the rate of PH (*p* = 0.037). In contrast, no significant association was observed for PTX (*p* = 0.64).

**TABLE 2:** Correlation between traversed parenchymal distance and complication types.

Complication		Distance (*N* = 226)				*p*
		2–4 cm	4–6 cm	6–8 cm	> 8 cm	
Pneumothorax	Yes	11	33	18	4	0.64^a^
	No	25	92	32	11	
Pulmonary hemorrhage	Yes	6	50	15	3	0.037^a^
	No	30	75	35	12	
Hemoptysis	Yes	0	2	1	0	0.818^a^
	No	36	123	49	15	
Other complications	Yes	0	1	1	0	0.759^a^
	No	36	124	49	15	

^a^ Using chi‐squared test.

When evaluating potential risk factors through multivariable logistic regression, the model identified two significant independent predictors for PTX: male gender, which was associated with a markedly higher risk (aOR = 3.78; 95% CI: 1.81–7.89; *p* < 0.001), and the number of biopsy samples, where an increase in samples was surprisingly linked to a decreased risk (aOR = 0.65; 95% CI: 0.45–0.94; *p* = 0.022). Notably, the traversed lung parenchymal distance did not emerge as a statistically significant independent predictor in this multivariable model (*p* = 0.251).

The results of the multivariable logistic regression analysis for PTX, detailed in Table 3, identified two statistically significant independent predictors. Men had a significantly higher risk of PTX compared with women (aOR = 3.78; 95% CI: 1.81–7.89; *p* < 0.001). An increase in the number of samples was associated with a decreased risk of PTX (aOR = 0.65; 95% CI: 0.45–0.94; *p* = 0.022). Traversed lung parenchymal distance was not a statistically significant independent predictor in this model (*p* = 0.251).

**TABLE 3:** Multivariable regression analysis to identify independent predictors for pneumothorax.

Predictor	aOR (95% KTC)	*p*
Age	1.01 (0.98–1.04)	0.451
Gender (male vs. female)	3.78 (1.81–7.89)	< 0.001
Number of biopsy samples	0.65 (0.44–0.94)	0.022
Lesion‐to‐pleura distance	1.10 (0.90–1.34)	0.251

Abbreviations: aOR = adjusted odds ratio; CI = confidence interval.

To address potential confounders and evaluate independent predictors for PH, a separate multivariable logistic regression analysis was conducted in Table 4. The model incorporated age, gender, quantity of biopsy samples, and traversed lung parenchymal distance. The analysis showed that the traversed lung parenchymal distance was not a statistically significant independent predictor of PH (aOR = 1.11; 95% CI: 0.92–1.34; *p* = 0.289). Interestingly, having more biopsy samples was linked to a lower risk of PH (aOR = 0.65; 95% CI: 0.46–0.93; *p* = 0.020). Other factors, such as female gender relative to male (aOR = 1.41; 95% CI: 0.77–2.58; *p* = 0.266) and age (aOR = 0.98; 95% CI: 0.95–1.00; *p* = 0.104), did not exhibit statistically significant associations in this multivariable model.

**TABLE 4:** Multivariable regression analysis to identify independent predictors for pulmonary hemorrhage.

Predictor	aOR (95% KTC)	*p*
Age	0.98 (0.95–1.00)	0.104
Gender (male vs. female)	1.41 (0.77–2.58)	0.266
Number of biopsy samples	0.65 (0.46–0.93)	0.020
Lesion‐to‐pleura distance	1.11 (0.92–1.34)	0.289

Abbreviations: aOR = adjusted odds ratio; CI = confidence interval.

## 4 Discussion

This study provides insight into the risk factors associated with complications of CT‐TTNB in the Vietnamese patient population. The overall complication rate in our study was 47.35%, with PTX (29.2%) and PH (32.74%) being the most common. These rates fall within the wide range reported in international literature, which notes PTX rates from 8% to 45% and PH rates from 3% to 55%, suggesting the consistency of our study's results with global data [4].

A key finding of this study is the role of the traversed lung parenchymal distance. While many previous studies identified this as a major risk factor for both PTX and PH [5], our study found no statistically significant independent association (*p* = 0.251 for PTX). This suggests that when adjusted for other factors, the impact of distance may not be as strong as univariate analyses have suggested.

In our univariable analysis, the traversed lung parenchymal distance showed a significant association with PH (*p* = 0.037) but not with PTX (*p* = 0.64). However, this significance disappeared in the multivariable model (*p* = 0.251). This transition suggests that the effect of distance might be attenuated or “absorbed” by other more dominant factors within our cohort, such as male gender. In the clinical context of Vietnam, male gender often serves as a proxy for chronic smoking and underlying lung diseases, which may fundamentally alter the lung's structural response to needle trauma regardless of the path length.

Instead of distance, our study identified male gender as a strong and independent predictor for PTX (aOR = 3.78). This finding is consistent with several previous studies [1, 6, 7]. The reason for this association may be that men have a higher prevalence of smoking and COPD, which are factors that reduce lung elasticity and increase the risk of air leakage. In the demographic context of Vietnam, male gender acts as a strong surrogate variable for chronic tobacco exposure. Recent epidemiological data indicates a massive gender disparity in tobacco use in Vietnam, with smoking prevalence exceeding 42.8% among men, compared with approximately 1.3% among women [8]. This prolonged exposure contributes to localized emphysema, alveolar destruction, and reduced pleural elasticity, which fundamentally alters the lung's mechanical response to needle trauma, explaining why gender eclipsed physical needle distance as a primary risk factor in our cohort. However, some other studies have not found an association between gender and the risk of PTX, indicating the need for further research to clarify this issue [4].

Another noteworthy finding is the inverse relationship between the number of biopsy samples and the risk of PTX (aOR = 0.65). This contradicts the assumption that more samples would cause more damage. However, this phenomenon can be explained by the “tamponade effect” of localized intraparenchymal bleeding. Repeated passes of the coaxial needle cause minor microvascular trauma, flooding the needle tract with blood. Because blood activates the coagulation cascade upon contact with extravascular tissue, it rapidly forms a biological mechanical seal (clot) within the tract. This clot effectively isolates the pleural space from the alveolar pressure gradient, neutralizing the air leak and providing a protective effect against PTX.

This physiological mechanism is supported by our univariable data, which indicated a significant association between the traversed parenchymal distance and PH (*p* = 0.037). As clinical observations suggest, the presence of limited, localized PH paradoxically serves as a protective factor against PTX by facilitating this clotting effect along the biopsy tract.

Interestingly, our multivariable analysis also revealed that a higher number of biopsy samples was associated with a significantly decreased risk of PH (aOR = 0.65, *p* = 0.020). Rather than implying that repetitive tissue cutting prevents bleeding, this finding is most likely a reflection of confounding by indication (operator selection bias). In clinical practice, if significant bleeding is observed by the interventional radiologist after the initial needle passes, the procedure is typically terminated prematurely to prioritize patient safety, resulting in a lower number of total samples collected in hemorrhagic cases. Conversely, during uncomplicated procedures with no immediate signs of bleeding, operators confidently proceed to obtain additional core samples to maximize diagnostic yield.

Furthermore, obtaining a sufficient number of samples in a single, well‐planned coaxial approach is safer than having to make multiple pleural punctures or repeat the procedure due to an inadequate sample. Our results align with a study by Ko et al. that showed that longer needle dwell time in the lung did not increase the risk of PTX and concluded that this should not deter from taking additional samples to ensure diagnosis [9, 10]. A more recent study of 1151 patients undergoing ultrasound‐guided biopsy also found no evidence of an association between the number of core biopsies and complications [11]. It is possible that obtaining a sufficient number of samples in a single, well‐planned approach is safer than having to make multiple needle adjustments or repeat the procedure due to an inadequate sample, thereby indirectly reducing the risk.

### 4.1 Limitations

Our study has some limitations. Due to its retrospective and single‐center design, the generalizability of the results may be limited. We also did not collect data on other important potential confounding factors such as the presence and severity of emphysema, the experience of the performing physician, or the type of needle used, all of which have been shown to influence complication rates in other studies [12].

Furthermore, as this is a cross‐sectional study, the identified predictors should be viewed as significant associations within our specific cohort rather than direct causal inferences. The high odds ratio observed in male patients likely reflects unmeasured respiratory comorbidities prevalent in this demographic.

## 5 Conclusion

Our study demonstrates that within this Vietnamese patient population, male gender and the number of biopsy samples are significantly associated with the risk of PTX. While the traversed lung parenchymal distance did not emerge as a standalone independent predictor in the multivariable analysis, it remains a critical parameter for procedural planning. These insights suggest that risk assessment should not rely on a single parameter but rather a multifactorial approach. Given the study's limitations in adjusting for all potential confounders such as emphysema, these results should be interpreted as clinical associations that warrant further validation through prospective, multicenter studies.

## Author Contributions

**Thanh Dinh Le:** project administration, conceptualization, methodology, writing – original draft, writing – review and editing. **Thuan Nguyen Huynh:** conceptualization, methodology, software, writing – review and editing, writing – original draft. **Thanh Chi Nguyen:** writing – original draft, writing – review and editing, validation. **Hau Thi Tran:** writing – review and editing, writing – original draft, visualization. **Nguyen Vo Cong Do:** resources, project administration, writing – original draft. **Khang Quang Le:** data curation, validation. **Son Thai Tran:** software, visualization. **Thanh Toan Vo:** writing – review and editing, writing – original draft, investigation, resources, supervision.

## Funding

The authors have nothing to report.

## Ethics Statement

This study complied with the ethical standards outlined by the Declaration of Helsinki. Ethical approval for this retrospective study was obtained from the Ethics Committee of Thong Nhat Hospital (Approval No. 738/BVTN 07/05/2025).

## Conflicts of Interest

The authors declare no conflicts of interest.

## Data Availability

The data that support the findings of this study are available on request from the corresponding author. The data are not publicly available due to privacy or ethical restrictions.
